# Structure of AadA from *Salmonella enterica*: a monomeric aminoglycoside (3′′)(9) adenyltransferase

**DOI:** 10.1107/S1399004715016429

**Published:** 2015-10-31

**Authors:** Yang Chen, Joakim Näsvall, Shiying Wu, Dan I. Andersson, Maria Selmer

**Affiliations:** a Department of Cell and Molecular Biology, Uppsala University, Biomedical Center, Box 596, SE-751 24 Uppsala, Sweden; b Department of Medical Biochemistry and Microbiology, Uppsala University, Biomedical Center, Box 582, SE-751 23 Uppsala, Sweden

**Keywords:** antibiotic resistance, aminoglycoside, X-ray crystallography, small-angle X-ray scattering

## Abstract

Aminoglycoside resistance is commonly conferred by enzymatic modification of drugs by aminoglycoside-modifying enzymes such as aminoglycoside nucleo­tidyltransferases (ANTs). Here, the first crystal structure of an ANT(3′′)(9) adenyltransferase, AadA from *Salmonella enterica*, is presented. AadA catalyses the magnesium-dependent transfer of adenosine monophosphate from ATP to the two chemically dissimilar drugs streptomycin and spectinomycin. The structure was solved using selenium SAD phasing and refined to 2.5 Å resolution. AadA consists of a nucleotidyltransferase domain and an α-helical bundle domain. AadA crystallizes as a monomer and is a monomer in solution as confirmed by small-angle X-ray scattering, in contrast to structurally similar homodimeric adenylating enzymes such as kanamycin nucleotidyltransferase. Isothermal titration calorimetry experiments show that ATP binding has to occur before binding of the aminoglycoside substrate, and structure analysis suggests that ATP binding repositions the two domains for aminoglycoside binding in the interdomain cleft. Candidate residues for ligand binding and catalysis were subjected to site-directed mutagenesis. *In vivo* resistance and *in vitro* binding assays support the role of Glu87 as the catalytic base in adenylation, while Arg192 and Lys205 are shown to be critical for ATP binding.

## Abstract 2

The crystal structure of the aminoglycoside-adenylating enzyme AadA is reported together with functional experiments providing insights into its oligomeric state, ligand binding and catalysis.

## 1. Introduction   

Ever since the discovery of the first aminoglycoside antibiotic, streptomycin (Schatz *et al.*, 1944 ▸), which was isolated from *Streptomyces griseus*, these broad-spectrum antibiotics have been widely used in the treatment of bacterial infections. Aminoglycosides have been shown to increase misreading and to inhibit translocation in bacterial translation as well as to damage the cell membrane (Davis, 1987 ▸). Crystal structures of aminoglycosides bound to the 30S ribosomal subunit (Brodersen *et al.*, 2000 ▸; Carter *et al.*, 2000 ▸; Demirci *et al.*, 2013 ▸) shed new light on their mechanisms of increasing errors in decoding. Streptomycin has been shown to stabilize the ribosomal ambiguity (*ram*) or error-prone state of the 30S subunit as well as to perturb the structure of the decoding centre, while other aminoglycosides such as paromomycin instead influence the conformations of the 16S rRNA bases directly involved in decoding (Carter *et al.*, 2000 ▸; Demirci *et al.*, 2013 ▸). Spectinomycin is an aminoglycoside-like aminocyclitol that inhibits translocation through preventing conformational changes of the head domain of the 30S subunit (Borovinskaya *et al.*, 2007 ▸).

Resistance to aminoglycosides can be acquired by four general mechanisms: reduction of the drug concentration in the cell by efflux pumps, decreased uptake of aminoglycosides into the cell *via* decreased cell-membrane permeability, alteration of the drug-binding site by mutation or chemical modification of the 16S RNA or ribosomal proteins, and enzymatic modification of aminoglycosides, leading to drug inactivation and diminished binding (Azucena & Mobashery, 2001 ▸; Davies & Wright, 1997 ▸; Llano-Sotelo *et al.*, 2002 ▸). Enzymatic modification of aminoglycosides is the most common mechanism of resistance observed clinically (Wright, 2011 ▸) and is mediated by three types of amino­glycoside-modifying enzymes (AMEs): aminoglycoside *O*-nucleotidyltransferases (ANTs), aminoglycoside *N*-acetyltransferases (AACs) and aminoglycoside *O*-phosphotransferases (APHs). The ANT family is the smallest and the least studied of the three. The ANT enzymes in general use ATP and magnesium to adenylate specific hydroxyl groups of their substrates, while some of them can also use other NTPs and/or other divalent ions. They are further classified depending on the site of substrate modification (Azucena & Mobashery, 2001 ▸; Jana & Deb, 2006 ▸), which is also linked to their substrate specificity. The ANT enzymes show low overall sequence identity, but have been suggested to share a similar fold required for ATP and Mg^2+^ binding (Wright, 1999 ▸). Crystal structures are available of the ANT(4′)-Ia kanamycin nucleotidyltransferase (PDB entry 1kny; Pedersen *et al.*, 1995 ▸; Sakon *et al.*, 1993 ▸), the ANT(4′)-IIb enzyme (PDB entries 4ebj and 4ebk; Center for Structural Genomics of Infectious Diseases, unpublished work), the ANT(6)-Ia enzyme (PDB entry 2pbe; New York SGX Research Center for Structural Genomics, unpublished work) and, recently, the ANT(2′′)-Ia enzyme (PDB entries 4wqk and 4wql; Cox *et al.*, 2015 ▸).

**Figure 1:**
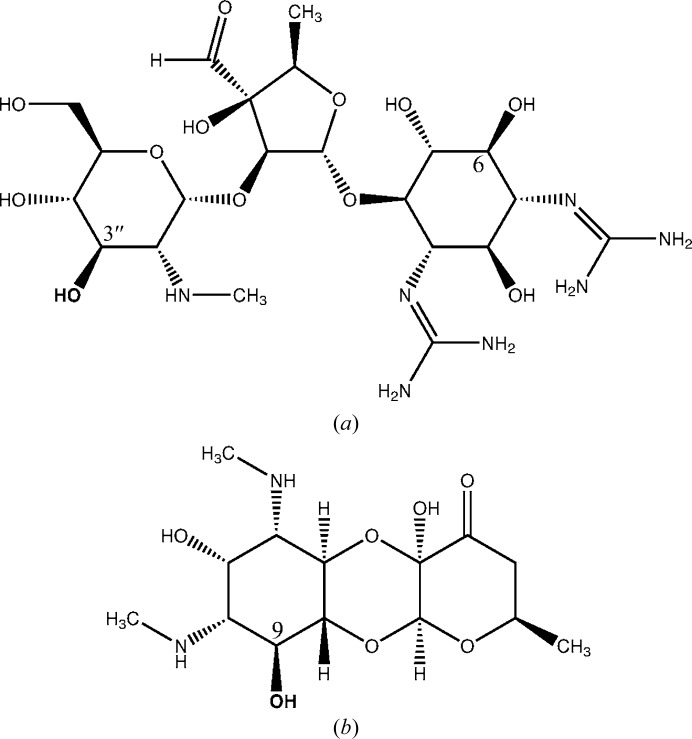
Aminoglycoside substrates of the ANT(3′′)(9) AadA. (*a*) Streptomycin with the adenylation-site 3′′-hydroxyl in bold. (*b*) Spectinomycin with the adenylation-site 9-hydroxyl in bold.

*Salmonella enterica* is a leading cause of foodborne and waterborne disease in humans and animals. Severe *Salmonella* infections are treated with antibiotics, but aminoglycosides are not usually considered as an option (Crump *et al.*, 2015 ▸). Still, the *S. enterica* genome contains an *aadA* gene (Hollingshead & Vapnek, 1985 ▸) encoding an ANT enzyme. AadA is an ANT(3′′)(9) streptomycin/spectinomycin adenyltransferase encoded by the *aadA* gene that adenylates the 3′′-hydroxyl group of the streptomycin glucosamine ring and the 9-hydroxyl group of the spectinomycin actinamine ring (Fig. 1 ▸). In this study, we present a structural and functional characterization of AadA from *S. enterica*, providing insights into its oligomeric state, ligand binding and catalysis.

## 2. Materials and methods   

### 2.1. Cloning of *aadA* into pEXP5-CT   

Bacterial strains and plasmids are listed in Supplementary Table S1. The *aadA* gene was PCR-amplified from a colony suspension of *S. enterica* serovar Typhimurium strain LT2 using Phusion High-Fidelity DNA Polymerase (Thermo Scientific) according to the manufacturer’s instructions with the primers aadA_start_Fwd and aadA_CT_Rev (Supplementary Table S2) and was cloned into the pEXP5-CT/TOPO vector (Invitrogen) according to the manufacturer’s protocol. Transformants were selected on LA plates supplemented with 100 mg l^−1^ ampicillin. Ampicillin-resistant transformants were screened for the correct insert by PCR and sequencing using the primers T7_Forward and T7_Term_Reverse (Supplementary Table S2). The resulting plasmid pEXP5-CT-*aadA* encodes the complete AadA sequence followed by a C-terminal linker and hexahistidine tag (KGHHHHHH).

### 2.2. Construction of *aadA* point mutations   

The eight point mutations in *aadA* were generated in two steps. A *cat-sacB*-T0 cassette (GenBank KM018298) containing the *cat* gene (conferring chloramphenicol resistance) and the *Bacillus subtilis sacB* gene (conferring sensitivity to sucrose) was inserted at the five target codons (codons 87, 112, 182, 192 and 205) using λ-Red recombineering (Datsenko & Wanner, 2000 ▸; Datta *et al.*, 2006 ▸), selecting chloramphenicol-resistant colonies. In the second step, a 70-mer oligonucleotide containing the designed mutation in the middle was used in a λ-Red transformation to replace the *cat-sacB*-T0 cassette, selecting sucrose-resistant cells that had lost the *sacB* gene. Sucrose-resistant, chloramphenicol-sensitive transformants were verified by PCR and sequencing of the *aadA* gene.

### 2.3. Gap-repair cloning of mutant *aadA* alleles   

To transfer the mutant *aadA* alleles from the chromosome to the pEXP5-CT-*aadA* plasmid, a gap-repair cloning strategy was used. The pEXP5-CT-*aadA* plasmid containing the wild-type *aadA* gene was linearized using StuI and PvuII (Thermo Scientific), which cut within the *aadA* gene. 5 ng of the linearized plasmid was used as template in a PCR reaction using Phusion DNA polymerase (Thermo Scientific) with the primers aadA_grc-r and aadA_grc-f (Supplementary Table S2). The resulting PCR product contains the entire pEXP5-CT vector sequence flanked by the first 116 bp of the *aadA* gene at one end and the last 68 bp at the other end (thus leaving a gap of 602 bp in *aadA*). After digestion with DpnI (Thermo Scientific) to remove any surviving nonlinearized template plasmid, the PCR fragment was transformed into strains with the mutant *aadA* alleles on the chromosome and the λ-Red recombineering system expressed from the pSIM5-Tet plasmid, selecting ampicillin-resistant cells that had repaired the pEXP5-CT-*aadA* plasmid through recombination with the chromosomal *aadA* gene.

### 2.4. MIC determinations (E-tests)   

Determination of the minimum inhibitory concentrations (MICs) of streptomycin, spectinomycin, amikacin, tobramycin, gentamicin and kanamycin were performed using E-tests (bioMérieux). As AadA is not expressed during growth on rich medium (Koskiniemi *et al.*, 2011 ▸), the tests were performed using minimal medium. Cultures grown overnight at 37°C in liquid M9 + 0.2% glycerol medium were diluted 500-fold and swabbed onto M9 + 0.2% glycerol agar plates using sterile cotton swabs. E-test strips were applied to the plates, which were incubated at 37°C for approximately 24 h. The MICs were read as the lowest concentration of antibiotic at which no bacterial growth was visible.

### 2.5. Expression and purification of AadA protein   

pEXP5-CT-*aadA* was transformed into *Escherichia coli* BL21 Star cells. To express native wild-type or mutant AadA protein, a 1 l culture in LB with 100 µg ml^−1^ ampicillin was inoculated with 10 ml overnight culture and incubated at 37°C until an OD_600_ of 0.6 was reached, and then chilled to 16°C before induction with 1 m*M* isopropyl β-d-1-thiogalactopyranoside (IPTG) for 24 h. Expression of selenomethionine-substituted AadA was performed according to a standard protocol (Van Duyne *et al.*, 1993 ▸). Cells were harvested by centrifugation and stored at −20°C.

All AadA variants were purified using the same protocol. Cells were resuspended in buffer *A* (50 m*M* Tris–HCl pH 7.5, 200 m*M* NaCl, 50 m*M* imidazole, 5 m*M* β-mercaptoethanol) containing DNase, RNase, lysozyme and cOmplete protease inhibitor (Roche) and were lysed using a cell disruptor (Constant Systems). After centrifugation in an SS34 rotor at 16 000 rev min^−1^ for 30 min, the supernatant was loaded onto a pre-equilibrated Ni Sepharose gravity column and incubated under slow rotation at 4°C for 1 h. The column was washed extensively with buffer *A* and with buffer *A* containing 500 m*M* NaCl, and AadA was eluted with buffer *B* (50 m*M* Tris–HCl pH 7.5, 200 m*M* NaCl, 500 m*M* imidazole, 5 m*M* β-mercaptoethanol). Protein-containing fractions were loaded onto a HiLoad 16/60 Superdex 75 gel-filtration column equilibrated with buffer *C* (50 m*M* Tris–HCl pH 7.5, 200 m*M* NaCl, 5 m*M* β-mercaptoethanol). Peak fractions were concentrated to 10 mg ml^−1^ and used directly for crystallization or stored at −80°C after shock-freezing in liquid nitrogen.

### 2.6. Crystallization   

Crystallization was performed using the sitting-drop method at 8°C. Crystals appeared in 24 h in the Morpheus screen (Molecular Dimensions) with a drop size of 2 µl and a reservoir solution consisting of 0.12 *M* alcohols, 0.1 *M* Morpheus buffer system 1 pH 6.5 and 30% ethylene glycol/PEG 8000. Most crystals grew as thin plates with dimensions of around 50 × 100 µm, while a few appeared as thin rods. Plate-shaped crystals were fished out directly from the drop and vitrified in liquid nitrogen for data collection.

### 2.7. Data collection and structure determination   

All data were collected on beamline ID14-4 at ESRF, Grenoble, France. Initial phases were obtained by single-wavelength anomalous diffraction (SAD) phasing using crystals of selenomethionine-substituted protein and data collected at the peak wavelength of 0.9793 Å, as determined by a fluorescence scan. Data were integrated and scaled using *XDS* (Kabsch, 2010 ▸) and *AIMLESS* (Evans & Murshudov, 2013 ▸) (Table 1 ▸) and suggested one molecule per asymmetric unit, with 51% solvent content and a Matthews coefficient of 2.53 Å^3^ Da^−1^ (Matthews, 1968 ▸).

Three Se sites were identified using *AutoSol* implemented in *PHENIX* (Adams *et al.*, 2010 ▸). The figure of merit was 0.37 (0.72 after density modification). An initial model with an *R*_work_ and *R*_free_ of 0.38 and 0.40, respectively, and 200 out of 262 residues (44 with unassigned sequence) was built and refined using *AutoBuild* in *PHENIX*. Further manual rebuilding was aided by *B*-factor map sharpening in *Coot* (Emsley *et al.*, 2010 ▸). A model consisting of 252 amino acids was built and refined to an *R*_work_ and *R*_free_ of 0.29 and 0.35, respectively, using the anomalous data. A 2.5 Å resolution native data set was subsequently collected, allowing completion of the model to 260 amino acids and refinement using *phenix.refine* (Afonine *et al.*, 2012 ▸) to an *R*_work_ and *R*_free_ of 0.23 and 0.26, respectively (Table 1 ▸). TLS refinement was implemented in the last round of refinement. Atomic coordinates and structure factors have been deposited in the Protein Data Bank with accession code 4cs6.

### 2.8. Structure analysis   

Detailed structure comparisons were performed using *SSM* in *Coot* (Emsley *et al.*, 2010 ▸) and the *LSQ* commands in *O* (Jones *et al.*, 1991 ▸; Kleywegt & Jones, 1997 ▸), which were also used as the basis for structure-based sequence alignment. Surface-conservation analysis was performed using the *ConSurf *server (Celniker *et al.*, 2013 ▸; Ashkenazy *et al.*, 2010 ▸). Structure figures were made using *PyMOL* (v.1.2r3pre, Schrödinger). Multiple sequence alignment was performed using *ClustalW* (Larkin *et al.*, 2007 ▸). The *EsPript* server (Gouet *et al.*, 2003 ▸) was used to prepare sequence-alignment figures.

### 2.9. Isothermal titration calorimetry (ITC) binding experiments   

Binding studies were performed at 25°C using a MicroCal iTC200 instrument (GE Healthcare). Wild-type or mutant AadA at 20–30 µ*M* concentration was dialyzed overnight against 50 m*M* Tris–HCl pH 7.5, 200 m*M* NaCl, 5 m*M* MgCl_2,_ 1 m*M* tris(2-carboxyethyl)phosphine and titrated with 500–1000 µ*M* ATP, streptomycin (Sigma) or spectinomycin (Sigma) freshly dissolved in the same batch of dialysis buffer prior to each experiment. For titration of streptomycin/spectinomycin in the presence of ATP, a first titration of AadA with ATP to saturation was followed by a second titration with streptomycin/spectinomycin in buffer containing an equivalent concentration of ATP as in the cell. In this way, heats of dilution of ATP were avoided. At least 36 consecutive injections of 2 µl were applied at 2 min intervals. The data were analyzed using the *MicroCal Analysis* plugin in *Origin*. All ITC data were analyzed assuming one set of binding sites. Each experiment was performed at least twice. For spectinomycin, the concentration used to fit the data was adjusted to the estimated active concentration in binding to AadA, assuming 1:1 binding.

### 2.10. SAXS measurements and analysis   

SAXS data were collected on beamline P12 at the PETRA synchrotron, EMBL, Hamburg, Germany (Blanchet *et al.*, 2015 ▸). For data collection, 1 mg ml^−1^ AadA was dialyzed against buffer *C* and was further concentrated to 10 mg ml^−1^. The concentration was determined using a Rudolph Research Analytical J357 refractometer.

Data were measured at concentrations of 1, 2, 5 and 10 mg ml^−1^ and normalized to the intensity of the transmitted beam, and the scattering of the buffer was subtracted. Data processing was performed using the *ATSAS* software package (Petoukhov *et al.*, 2012 ▸). Theoretical scattering curves were calculated from PDB coordinates and fitted to the experimental scattering curve at 5 mg ml^−1^ concentration using *CRYSOL* (Svergun *et al.*, 1995 ▸). The radius of gyration was computed using *GNOM* (Svergun, 1992 ▸). The molecular weight was estimated from the Porod volume using bovine serum albumin as a standard.

## 3. Results and discussion   

### 3.1. Structure determination of AadA   

AadA crystals grew in space group *P*2_1_2_1_2 and diffracted to 2.5 Å resolution (Table 1 ▸). Trials to perform molecular replacement using search models with low sequence identity failed, and the structure was solved using SAD phasing with selenomethionine-substituted AadA crystals. In order to assist in model building, a *DALI* search (Holm & Rosenström, 2010 ▸) was performed with the incomplete autobuilt N-terminal domain. The top hit, with an r.m.s.d. of 2.6 Å over 96 C^α^ atoms, was a hypothetical protein from *Haemophilus influenzae* (PDB entry 1no5; Lehmann *et al.*, 2005 ▸) classified as a nucleotide-binding domain of a two-protein nucleotidyltransferase. Although this structure was not successful as a molecular-replacement search model, the connectivity was similar and it could be used to guide the manual building of the remaining parts of the N-terminal domain of AadA. The final model contains residues 3–262 and only lacks the first two N-terminal residues and the His tag. The loop regions 97–103 and 235–240 display weak density, indicating flexibility.

### 3.2. Overall structure of AadA   

**Figure 2:**
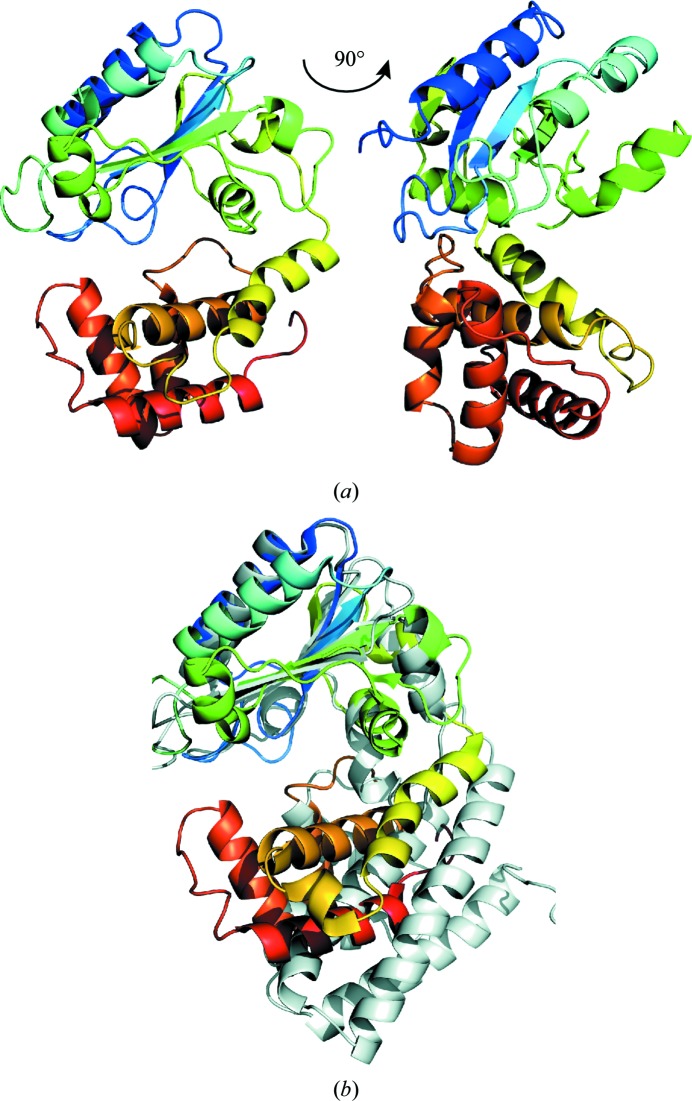
(*a*) Overall structure of AadA in rainbow colours from the N-terminal end in blue to the C-terminal end in red. The two views are 90° apart. (*b*) Superposition of AadA and KNTase (PDB entry 1kny; Pedersen *et al.*, 1995 ▸) based on the N-terminal domain. KNTase is shown in grey.

AadA consists of two domains that together form a bi-lobed 55 × 40 × 35 Å structure (Fig. 2 ▸ *a*). The N-terminal domain (residues 3–157) forms a nucleotidyltransferase fold according to the SCOP database (Murzin *et al.*, 1995 ▸) and has a central five-stranded mixed β-sheet surrounded by six α-helices. The β-strands β2 and β3 are parallel and the others are antiparallel. The long α-helices α1, α2, α4 and α5 surround the central β-sheet, whereas α3 is a single-turn helix following β3 and α6 is another short helix that follows β5 before continuing to the C-terminal domain. The C-terminal domain (residues 158–262) consists of five α-helices forming an up-and-down α-helical bundle.

### 3.3. Comparison of AadA to similar structures   

A search for similar structures in the PDB was performed using the *DALI* server (Holm & Rosenström, 2010 ▸). In a *DALI* search with the entire AadA molecule, the top hit, with a *Z*-score of 9.5, was the ANT(4′) kanamycin nucleotidyltransferase (KNTase) from *S. aureus* (PDB entry 1kny; Pedersen *et al.*, 1995 ▸; r.m.s.d. of 4.8 Å for 187 C^α^ atoms), which showed 14% amino-acid sequence identity to AadA. Several hypothetical predicted nucleotidyltransferase proteins also show similarities to AadA, with *Z*-scores from 8.9 to 6.7. Among these, the most similar is the hypothetical protein HI0073 (PDB entry 1no5; Lehmann *et al.*, 2005 ▸), which was used as a guide for building the N-terminal domain of AadA. The lincosamide nucleotidyltransferase LinB from *Entero­coccus faecium* (PDB entry 3jz0; Morar *et al.*, 2009 ▸) shows a lower similarity to AadA, with a *Z*-score of 6.7 and an r.m.s.d. of 5.6 Å for 173 C^α^ atoms, with only 9% sequence identity. Other available ANT structures displayed low structural similarity to AadA. Of the *DALI* hits, *S. aureus* KNTase (Chen-Goodspeed *et al.*, 1999 ▸; Pedersen *et al.*, 1995 ▸) and *E. faecium* LinB (Morar *et al.*, 2009 ▸) were biochemically characterized as nucleotidyltransferases acting on drug substrates, and structures were available in complex with the ATP analogue AMPCPP and the drug substrate. Further comparisons were performed with these two structures, both of which consist of N-terminal nucleotidyltransferase domains and C-terminal helical bundle domains. However, both of these proteins crystallize as homodimers, and the orientation between the two domains is distinct from that in AadA. Careful domain-by-domain superpositioning between AadA and KNTase shows that in the N-terminal domain helix α1 and the central β-sheet formed by β1–β5 of AadA superpose well on their equivalent secondary-structure elements in KNTase (r.m.s.d. of 1.59 Å for 81 C^α^ atoms; Fig. 2 ▸ *b*), and in the C-terminal domain helices α8–α11 of AadA superpose on their equivalents (r.m.s.d. of 2.23 Å for 57 C^α^ atoms; Supplementary Fig. S1). In the superpositioning of AadA with LinB, in the N-terminal domain helix α1 and strands β1–β3 from AadA superpose on LinB (r.m.s.d. of 2.05 Å for 73 C^α^ atoms) and in the C-terminal domain helices α7, α8 and α11 superpose on LinB (r.m.s.d. of 2.02 Å for 49 C^α^ atoms). Helices α2 and α4 in the N-terminal domain of AadA do not have any equivalents in the KNTase or LinB structures. Also, the connectivity of β2 and β3, as well as β4 and β5, in AadA is distinct from the other two structures.

### 3.4. Oligomeric state of AadA   

#### 3.4.1. Structure comparison with KNTase and LinB   

Both KNTase and LinB function as homodimers with very similar dimerization contacts and two active sites located at the dimer interfaces. *S. aureus* KNTase was believed to be monomeric from an early study using native gels (Sadaie *et al.*, 1980 ▸). Yet, the protein purified using the same procedure crystallized as a homodimer. Each monomer has a very extended conformation, and the binding pockets for kanamycin and ATP are formed by both subunits (Supplementary Fig. S2*a*). *S. enterica* AadA was purified as a monomer in size-exclusion chromatography and crystallized as a monomer. It has a more closed domain arrangement compared with KNTase (Fig. 2 ▸ *b*) and more extensive interdomain interactions within the monomer. In KNTase, the conserved ligand-binding surface displays a negative charge, whereas the conserved dimerization surface is uncharged (Supplementary Fig. S2*b*). Could the AadA structure potentially open up to also form a homodimer with two active sites? We find this unlikely for two reasons. Firstly, the surface corresponding to the dimer interface in KNTase is negatively charged and nonconserved, making it unlikely to be involved in the formation of a homodimer. Secondly, the highly conserved helix η2 preceding α4 in AadA that has no equivalent in KNTase (see below) would clash with the packing of helix α6 in the C-terminal domain of KNTase against the β-sheet of the N-terminal domain. Thus, we propose that AadA represents an ANTase that functions as a monomer.

#### 3.4.2. SAXS studies of AadA in solution   

**Figure 3:**
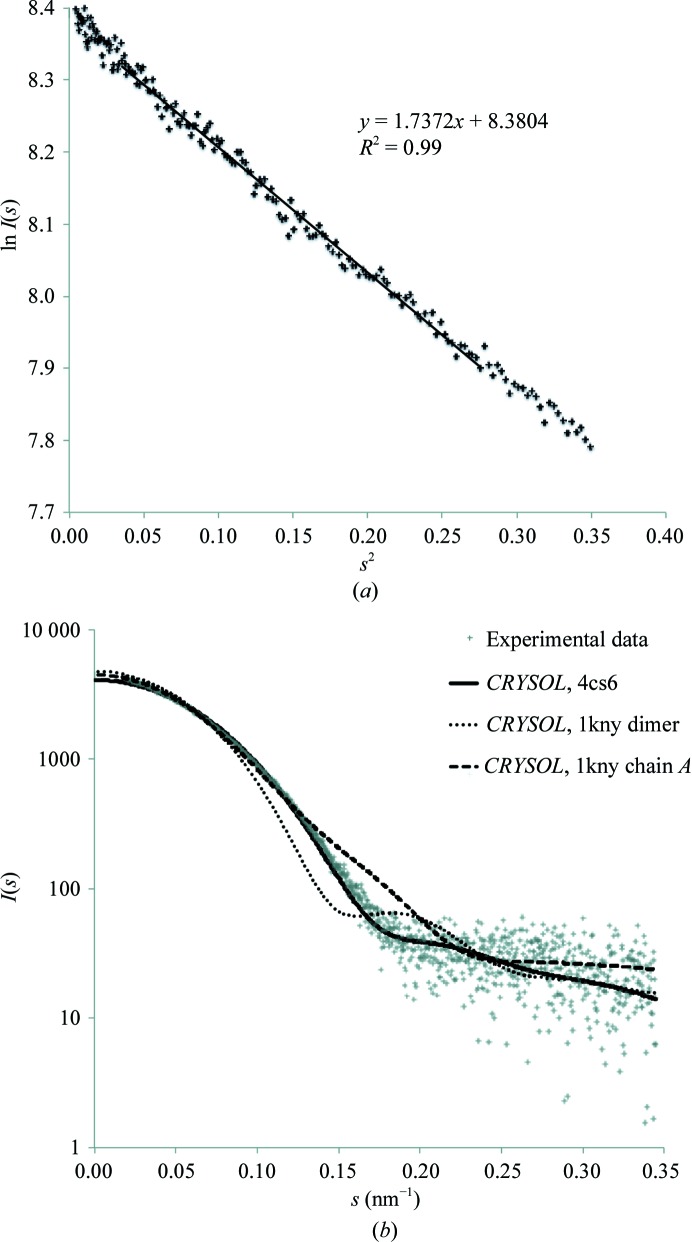
SAXS data. (*a*) Guinier plot showing the linear fit used to derive *R*_g_. (*b*) Experimental data overlaid with calculated scattering curves from PDB entries 4cs6 (AadA), 1kny (KNTase dimer; Pedersen *et al.*, 1995 ▸) and 1kny chain *A* (KNTase monomer; Pedersen *et al.*, 1995 ▸).

To confirm the oligomeric state of AadA in solution, we performed a SAXS experiment on apo AadA. There were no signs of concentration-dependent protein aggregation at 1–5 mg ml^−1^ concentration. The resulting Guinier plot was linear, consistent with a monodisperse protein preparation (Fig. 3 ▸ *a*). The molecular mass calculated from the SAXS data was 29 kDa. Comparison of the experimental SAXS data with the scattering curve predicted from the crystal structure (Fig. 3 ▸ *b*) gave an excellent fit with a χ value of 1.9, while the predicted scattering curves from the KNTase monomer and dimer (PDB entry 1kny) gave poor fits, with χ values of 11.3 and 35.5, respectively. The radius of gyration (*R*_g_) determined from the Guinier plot was 23 Å, which agrees well with the *R*_g_ of 20 Å calculated from the AadA crystal structure. Thus, the SAXS data confirm that AadA is indeed a monomer in solution and that the crystal structure agrees well with the solution structure.

### 3.5. Multiple sequence alignment   

**Figure 4:**
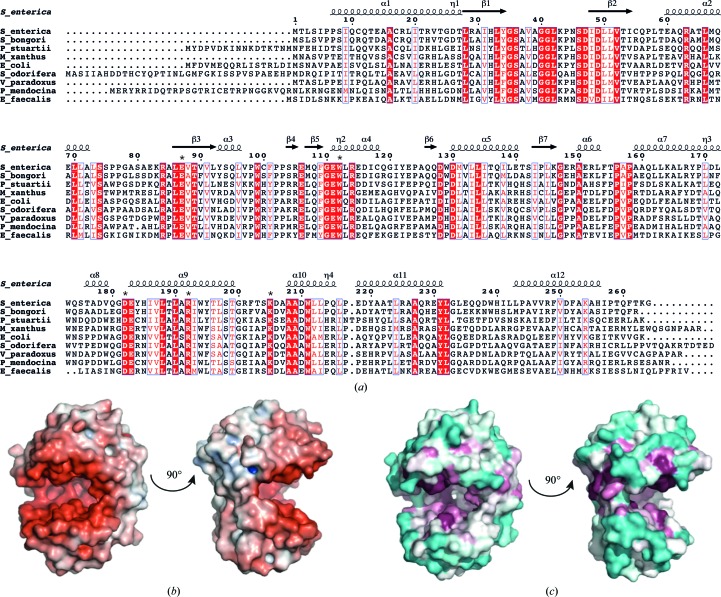
(*a*) Sequence alignment of *S. enterica* AadA with a representative set of homologues from different bacteria retrieved from a *BLAST* search (UniProt accession numbers are given in parentheses): *Salmonella bongori* (F8VG00), *Providencia stuartii* (I0E075), *Myxococcus xanthus* (Q1DBM4), *Escherichia coli* (C4NV14), *Serratia odorifera* (D4E6F0), *Variovorax paradoxus* (C5CZ95), *Pseudomonas mendocina* (A4XUC2) and *Enterococcus faecalis* D6 (C7UVI9). Strictly conserved residues are highlighted in red and conservative substitutions are in red font. The secondary structure of *S. enterica* AadA is shown above the alignment. The residues subjected to mutagenesis are indicated with asterisks above the alignment. (*b*) Electrostatic surface potential of AadA. The colour spectrum ranges from deep red (−7*kT*) to deep blue (+7*kT*). Orientations are as in Fig. 2 ▸(*a*). (*c*) Surface conservation mapped by *ConSurf* (Celniker *et al.*, 2013 ▸; Ashkenazy *et al.*, 2010 ▸). The colour spectrum ranges from magenta (highest conservation) to cyan (lowest conservation). Orientations are as in Fig. 2 ▸(*a*).

A *BLAST* search of the UniRef90 database identified full-length homologues of *S. enterica* AadA mainly in entero­­bacteria, proteobacteria and firmicutes. A representative set of these, displaying 36–81% sequence identity to the search sequence, was used for multiple sequence alignment (Fig. 4 ▸ *a*). In the N-terminal domain, residues involved in the hydrophobic packing between the β-sheet and the four long helices are well conserved, and the same is true for the interface between helices α8, α9 and α10 in the C-terminal domain. Among the conserved residues in AadA, Ser36 and Asp47 in the β1–β2 loop, Asp49 in β2, Glu87 and Thr89 in β3, Trp112 in α4, Asp182 and Arg192 in α9 and Lys205 in the α9–α10 loop are exposed at the surface and point towards the interdomain space.

### 3.6. Ligand-binding and catalytic sites of AadA   

We attempted without success to co-crystallize and soak AadA with its substrates streptomycin or spectinomycin together with the nonhydrolysable ATP analogue AMPCPP and magnesium. AadA has a pI of 5 and the electrostatic surface potential shows that the cleft between the two domains displays a strong negative charge (Fig. 4 ▸ *b*). Mapping of surface conservation using *ConSurf* (Ashkenazy *et al.*, 2010 ▸; Celniker *et al.*, 2013 ▸) shows that this is also where the AadA sequence displays the highest conservation (Fig. 4 ▸ *c*; see below). The negative charge may mimic the nucleic acid environment that the aminoglycosides bind to in the ribosome (Romanowska *et al.*, 2013 ▸) and is required for binding of the positively charged drug molecules.

#### 3.6.1. Comparative analysis of ligand-binding sites   

The structure of KNTase has been solved in the presence of kanamycin A (Pedersen *et al.*, 1995 ▸) and the structure of the more distantly related LinB has been solved in complex with clindamycin (Morar *et al.*, 2009 ▸), in both cases in the presence of AMPCPP and magnesium, thus allowing comparison of these structures with the apo structure of AadA. In both of these structures the ligands bind between the N-terminal domain of one monomer and the C-terminal domain of the second monomer in the dimer.

**Figure 5:**
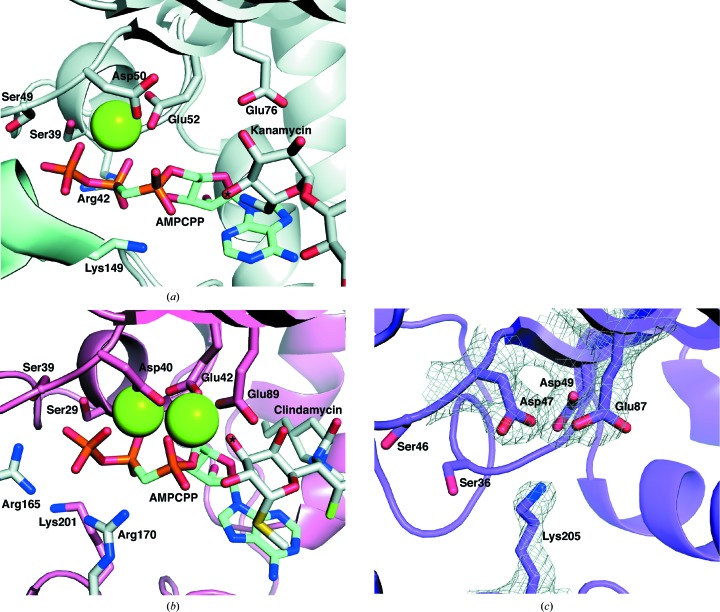
Nucleotide-binding sites of KNTase (*a*), LinB (*b*) and AadA (*c*). All figures are in the same orientation, according to superpositions based on the N-­terminal domain. Mg^2+^ ions are shown as green spheres. A 2*F*_o_ − *F*_c_ map for Asp47, Asp49, Glu87 and Lys205 in AadA is shown in (*c*) contoured at 1σ (0.23 e Å^−3^). The equivalent residues in KNTase and LinB are shown in (*a*) and (*b*). The adenylation sites of kanamycin and clindamycin are indicated by asterisks. The two monomers of KNTase (*a*) are shown in grey and pale blue and the two monomers of LinB (*b*) are shown in salmon and grey.

In KNTase, residues from both subunits make up the nucleotide-binding site (Fig. 5 ▸ *a*). From one subunit, Ser39 and Ser49 coordinate to the γ-phosphate of AMPCPP, Arg42 forms hydrogen bonds to the β-phosphate of AMPCPP and the ribose, Asp50 and Glu52 coordinate to the Mg^2+^ ion and Thr187 forms a hydrogen bond with the β-phosphate. From the other subunit, Glu145 and Lys149 form hydrogen bonds to the α-phosphate of AMPCPP. KNTase can use also other nucleotides as substrates, and there is no specific interaction between the KNTase and the base (Pedersen *et al.*, 1995 ▸). The nucleotide-binding pocket of LinB is very similar to that of KNTase (Fig. 5 ▸ *b*) and is formed by Ser29, Ser39, Asp40, Glu42 and Glu89 from one subunit and Arg165 and Arg170 from the other subunit (Morar *et al.*, 2009 ▸). The phosphate-coordinating serine residues and the magnesium-chelating acidic residues in KNTase and LinB (Morar *et al.*, 2009 ▸; Pedersen *et al.*, 1995 ▸; Figs. 5 ▸ *a* and 5 ▸ *b*) are conserved in AadA, where the corresponding residues that are likely to adopt the same roles are Ser36, Ser46, Asp47, Asp49 and Glu87 (Fig. 4 ▸ *c*). Apart from these residues, there is almost no sequence conservation between AadA and LinB. Therefore, structure-based sequence alignment (Supplementary Fig. S3) was only performed between AadA and KNTase.

In KNTase, Glu145 from the second subunit was proposed to be the catalytic base (Pedersen *et al.*, 1995 ▸), but there is no structurally equivalent residue in AadA (Supplementary Fig. S3). In LinB Glu89 was proposed to be the catalytic base, and it was confirmed by mutagenesis that this residue is essential for catalysis (Morar *et al.*, 2009 ▸). The equivalent residue in AadA, Glu87, is strictly conserved (Fig. 4 ▸ *a*), making it a good candidate for the catalytic base. In KNTase, this corresponds to Glu76, which is located in close proximity to the substrate kanamycin but does not have a clear role. However, in the overlay based on the N-terminal domain, the carboxylic O atoms of Glu145 of KNTase are within 2.5–5 Å of those of Glu87 in AadA, suggesting that these residues may play the same role in these enzymes, catalyzing the same reaction at different positions of different substrates. The active-site comparison (Fig. 5 ▸) suggests that Lys205 may be available for interaction with the phosphates of ATP. The remaining strictly conserved exposed residues in the AadA homologues (Thr89, Trp112, Asp182 and Arg192) are not conserved in KNTase or LinB. Therefore, these residues are more likely to be responsible for substrate interactions and specificity.

In the AadA structure, several of the residues predicted to participate in ATP binding are involved in interactions with the C-terminal domain: Ser36 forms a hydrogen bond to Asp206, Ser46 hydrogen-bonds to the amino group of Lys43, and Asp47 and Asp49 form salt bridges with Lys205. Thus, in the conformation observed in the apo AadA structure the nucleotide-binding site is blocked by interdomain inter­actions.

#### 3.6.2. *In vitro* ligand-binding studies using ITC   

**Figure 6:**
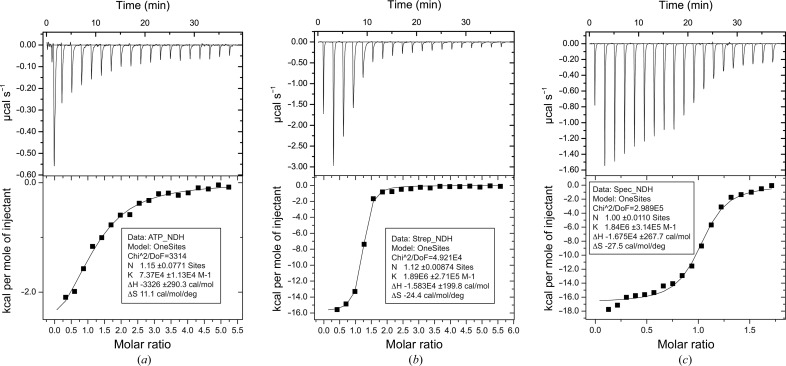
ITC curves for the titration of (*a*) ATP, (*b*) streptomycin and (*c*) spectinomycin with wild-type AadA. The top panels show the raw data and the bottom panels show the binding isotherms.

To test the binding affinity of ATP and aminoglycoside ligands for AadA, ITC experiments were performed (Table 3). We observed that ATP binds to wild-type AadA with a *K*_d_ of 13 µ*M* (Fig. 6 ▸ *a*), while the nonhydrolysable ATP analogue AMPCPP did not show any detectable binding. This indicates that in contrast to the observations for KNTase and LinB, an interaction with the O atom linking the α- and β-phosphates of ATP may be essential for ATP binding in AadA. Streptomycin and spectinomycin did not display detectable binding to AadA in the absence of ATP (data not shown). In the presence of ATP, both streptomycin and spectinomycin bind to AadA with an estimated *K*_d_ of 0.5 µ*M* (Figs. 6 ▸ *b* and 6 ▸ *c*). ATP and streptomycin showed binding to AadA with an approximate 1:1 stoichiometry. While the spectinomycin was sold as having a potency of 603 µg mg^−1^ (as estimated from a bacterial growth assay), the fit of the spectinomycin ITC data showed that its activity in binding to AadA was only 31% of the assumed active concentration. The data only indicate one type of binding site and there is no indication that AadA or any related adenyltransferase would have a different stoichiometry to one ATP and one adenylation substrate per enzyme. Therefore, the spectinomycin concentration was adjusted to 31% of the concentration based on the dry weight of the powder to fit the data, yielding an *N* value of 1. We do not have a clear chemical explanation for why only about half of the potent spectinomycin molecules bind to AadA, but it could possibly be related to the carbonyl–diol equilibrium of the drug in aqueous solution (Bryskier, 2005 ▸).

These results indicate that binding of ATP and magnesium between the two domains of AadA will orient the two domains for binding of either of the aminoglycoside substrates to the intersubunit pocket and that the O atom between the α- and β-phosphates forms a critical interaction with AadA. Thus, AadA binds ATP before the aminoglycoside substrate, in contrast to KNTase, where kanamycin first binds to a lower affinity nonspecific binding site and then relocates to the final binding cleft when a nucleotide is present (Matesanz *et al.*, 2012 ▸). This also agrees with the pre-formed ATP-binding site in the dimeric apo KNTase structure and the closed ATP-binding site of the present apo AadA structure. It is most likely that ATP binding will induce an open conformation of the structure in which residues from the two domains are correctly positioned for substrate recognition.

### 3.7. Mutational studies of AadA   

To test our hypotheses about the roles of the conserved amino acids Glu87, Trp112, Asp182, Arg192 and Lys205 in ligand binding and catalysis, we generated the following AadA mutants by mutating the chromosomal *aadA* gene: E87A, E87Q, W112A, W112F, D182A, D182N, R192A and K205A.

#### 3.7.1. *In vivo* functional tests   

We tested the effects of the individual mutations on the function of AadA by determining the minimal inhibitory concentrations (MICs) of streptomycin and spectinomycin (Table 2 ▸). Consistent with a direct role of Glu87 in catalysis, both of the generated mutants of amino acid 87 reduced the MIC to that of an *aadA* null strain (*aadA*::*cat*). Of the two mutations in position 112, the alanine substitution had a more severe effect than the phenylalanine substitution on the MIC of streptomycin, while both mutations reduced the MIC of spectinomycin to that of an *aadA* null strain. The mutations at positions 182, 192 and 205 reduced both MICs. The R192A and K205A mutants had MICs that were indistinguishable from that of the *aadA* null mutant, while both mutations at position 182 resulted in reduced MICs that were still higher than that of the *aadA* null strain. These results are consistent with roles of Trp112 in substrate binding and specificity and of Asp182, Arg192 and Lys205 in the binding of cofactor or substrate. However, these results cannot distinguish general effects such as misfolding or instability from specific effects such as loss of catalytic function or binding affinity.

#### 3.7.2. *In vitro* ligand-binding studies of mutants using ITC   

The effect of mutations on ligand binding was tested in ITC experiments (Table 3 ▸). E87Q and E87A displayed a threefold to fourfold lower affinity for ATP compared with the wild type, consistent with a role of the deprotonated Glu87 in magnesium-mediated coordination of the α-phosphate of ATP. For the R192A and K205A mutants no ATP binding could be detected, suggesting critical roles of these residues in the coordination of ATP. Comparison with KNTase and LinB suggests that Lys205 could form an interaction with the phosphates (Fig. 5 ▸).

The Glu87 mutants had a 20–40-fold lowered affinity for streptomycin and displayed no measurable binding of spectinomycin, agreeing with a direct or magnesium-mediated role of Glu87 in substrate coordination. Surprisingly, the W112F mutant did not affect the affinity for ATP or substrates, suggesting that the *in vivo* observation may be owing to effects on folding or on the correct orientation of the substrate for modification. The D182N mutation only diminished the binding of spectinomycin, suggesting a direct interaction. While the R192A mutant had a dramatically lowered affinity for both substrates, the K205A mutant could still bind both substrates, suggesting that this mutation either made the enzyme capable of binding the substrate in the absence of ATP or capable of binding substrate and ATP at the same time.

#### 3.7.3. Recognition of different adenylation substrates of AadA   

Streptomycin and spectinomycin are chemically very different, yet both are substrates of AadA and bind to the wild-type enzyme with similar affinities. What is common to the two modification sites? Both involve hydroxyl groups at positions next to a methylamine group on six-membered rings (Fig. 1 ▸), suggesting that this part of the two substrates could form similar interactions with the enzyme. At present we do not know whether the enzyme specifically binds these two molecules or whether the enzyme binds a broader range of aminoglycosides but only positions the appropriate hydroxyl groups of these two molecules for adenylation, in analogy with what has been described for the aminoglycoside-2′′-phosphotransferase family (Young *et al.*, 2009 ▸).

Expression of the *aadA* gene in *S. enterica* is only turned on under certain environmental conditions and is positively regulated by the stringent response regulator (p)ppGpp (Koskiniemi *et al.*, 2011 ▸). The presence of the *aadA* gene in the genome of *S. enterica*, a species that to our knowledge has not been under selection for aminoglycoside resistance (Crump *et al.*, 2015 ▸), suggests that there may also be an alternative adenylation substrate in the cell that remains to be identified.

## 4. Conclusions   

We have presented the first crystal structure of an ANT(3′′)(9) adenyltransferase: AadA from *S. enterica*. The crystal structure together with SAXS data shows that in contrast to the structurally similar kanamycin nucleotidyltransferase, AadA functions as a monomer in magnesium-dependent adenyl transfer.

We have shown using ITC that ATP binds to AadA before the aminoglycoside substrate and positions the two domains for aminoglycoside binding in the interdomain cleft. Candidate residues for ligand binding and catalysis were subjected to site-directed mutagenesis and assayed for effects on resistance *in vivo* and ligand binding *in vitro*. The assays support a role for Glu87 as the catalytic base in adenylation, while Arg192 and Lys205 are critical for ATP binding and Asp182 is more important for the binding of spectinomycin than streptomycin. The details of substrate binding and catalysis remain to be clarified in future studies.

## Supplementary Material

PDB reference: AadA, 4cs6

Supporting online material. DOI: 10.1107/S1399004715016429/mn5096sup1.pdf

## Figures and Tables

**Table 1:** Data-collection and refinement statistics Values in parentheses are for the highest resolution bin.

	AadA (SeMet)	AadA (native)†
Data collection		
Space group	*P*2_1_2_1_2	*P*2_1_2_1_2
Unit-cell parameters (, )	*a* = 49.0, *b* = 104.6, *c* = 58.8, = = = 90	*a* = 48.6, *b* = 104.6, *c* = 58.6, = = = 90
Resolution range ()	20.02.7 (2.802.70)	39.02.5 (2.652.50)
Wavelength ()	0.9793	1.000
Unique reflections	14255	10853
Completeness (%)	99.1 (100)	99.7 (98.5)
Multiplicity	3.77 (3.87)	7.05 (7.14)
*R*_meas_ (%)	8.1 (70.6)	8.1 (94.7)
*I*/(*I*)	12.6 (2.0)	17.0 (2.1)
Refinement		
Resolution range ()		39.02.5
Reflections (test set)		522
No. of atoms		
Protein		2052
Water		36
*R*_work_/*R*_free_ (%)		22.6/26.6
Average *B* factor (^2^)		57.3
R.m.s.d. from ideal		
Bond lengths ()		0.005
Bond angles ()		1.09
Ramachandran plot		
Preferred (%)		95.4
Allowed (%)		3.5
Outliers (%)		1.2

† Friedel pairs were merged for the native data set.

**Table 2:** Minimum inhibitory concentrations (MICs) of aminoglycoside antibiotics for strains with wild-type or mutant AadA nd, not determined.

		MIC† (gml^1^)					
Strain	*aadA* genotype/amino-acid substitution	Streptomycin	Spectinomycin	Kanamycin	Tobramycin	Amikacin	Gentamicin
DA6192	Wild type	128	192	6	3	4	0.75
DA18900	*aadA*::*cat* ‡	4	24	6	3	4	0.75
DA29580	E87A	4	24	6	nd	nd	nd
DA29582	E87Q	4	24	6	nd	nd	nd
DA29584	W112A	16	24	6	nd	nd	nd
DA29586	W112F	48	24	6	nd	nd	nd
DA29588	D182A	32	48	6	nd	nd	nd
DA29590	D182N	24	48	6	nd	nd	nd
DA29592	R192A	6	24	6	nd	nd	nd
DA29594	K205A	4	24	6	nd	nd	nd

† MICs were determined with E-tests (bioMrieux). All culture media were M9 plus 0.2% glycerol.

‡ The *cat* cassette replaced the entire *aadA* gene.

**Table 3:** Binding affinities of ATP and substrate ligands for AadA as determined by ITC nb, no binding detected.

AadA variant	*K*_d_(ATP) (*M*)	*K*_d_(streptomycin) (*M*)	*K*_d_(spectinomycin) (*M*)
Wild type	13 2	0.5 0.1	0.5 0.1
E87A	40 10	20 2	nb
E87Q	50 10	11 2	nb
W112F	20 5	0.8 0.1	0.6 0.1
D182N	16 3	0.7 0.1	>30
R192A	nb	>100	>30
K205A	nb	1.3 0.2	>10
