# Supplementary material for: Structure of AadA from Salmonella enterica: a monomeric aminoglycoside (3′′)(9) adenyltransferase
Source: Acta Crystallogr D Biol Crystallogr. 2015 Oct 31;71(Pt 11):2267–77. doi: 10.1107/S1399004715016429 (PMC4631478; doi:10.1107/S1399004715016429)
Supplement: Supplementary file 1 [file d-71-02267-sup1.pdf]

BIOLOGICAL
CRYSTALLOGRAPHY

Volume 71 (2015)

Supporting information for article:

Structure of AadA from *Salmonella enterica*: a monomeric aminoglycoside (3″)(9) adenyltransferase

Yang Chen, Joakim Näsvall, Shiying Wu, Dan I. Andersson and Maria Selmer

Table S1 Bacterial strains and plasmids. All strains are derivatives of *Salmonella enterica serovar Typhimurium* strain LT2.

Strain	Genotype	Source/Reference
DA6192	Wild type	Lab collection
DA18900	aadA::cat	(3)
DA27238	Wild type / pSIM5-Tet	Lab collection
DA28773	galE::cat-sacB-T0 (template for amplifying cat-sacB-T0)	Lab collection
DA29570	aadA87::cat-sacB-T0 / pSIM5-Tet	This study
DA29572	aadA112::cat-sacB-T0 / pSIM5-Tet	This study
DA29574	aadA182::cat-sacB-T0 / pSIM5-Tet	This study
DA29576	aadA192::cat-sacB-T0 / pSIM5-Tet	This study
DA29578	aadA205::cat-sacB-T0 / pSIM5-Tet	This study
DA29580	aadA(E87A)	This study
DA29582	aadA(E87Q)	This study
DA29584	aadA(W112A)	This study
DA29586	aadA(W112F)	This study
DA29588	aadA(D182A)	This study
DA29590	aadA(D182N)	This study
DA29592	aadA(R192A)	This study
DA29594	aadA(K205A)	This study
DA30831	aadA[E87A] / pSIM5-Tet	This study
DA30833	aadA[E87Q] / pSIM5-Tet	This study
DA30835	aadA[W112A] / pSIM5-Tet	This study
DA30837	aadA[W112F] / pSIM5-Tet	This study
DA30839	aadA[D182A] / pSIM5-Tet	This study
DA30841	aadA[D182N] / pSIM5-Tet	This study
DA30843	aadA[R192A] / pSIM5-Tet	This study
DA30845	aadA[K205A] / pSIM5-Tet	This study

Plasmid	Description	Source/Reference
pSIM5-Tet	Temperature inducible λ-red system	(3)
pEXP5-CT-aadA		This study
pEXP5-CT-aadA[E87A]		This study
pEXP5-CT-aadA[E87Q]		This study
pEXP5-CT-aadA[W112A]		This study
pEXP5-CT-aadA[W112F]		This study
pEXP5-CT-aadA[D182A]		This study
pEXP5-CT-aadA[D182N]		This study
pEXP5-CT-aadA[R192A]		This study
pEXP5-CT-aadA[K205A]		This study

Table S2 Oligonucleotides used in this study.

Primers for cloning and sequencing aadA in pEXP5-CT:	
aadA_start_Fwd	ATGACGCTGTCCATACCGCC
aadA_CT_Rev	TGTGAACTGCGTGGGGATGT
T7_Forward	TAATACGACTCACTATAGGG
T7_Term_Reverse	ATCCGGATATAGTTCCTCCTTTC
Primers for PCR amplification of the cat-sacB-T0 cassette with 40 bp flanking homologies to the five target codons in aadA[a]:	
aadA87-P1	TTCATCGCCGCCGGGGGCGTCGGCAGAAAAACGCGCTCTGTGTAGGCTGGAGCTGCTTC
aadA87-P2	AACACCAGGGAACCAGCTGCGAATAAAGCACGACAGTGACATATGAATATCCTCCTTAGTTCC
aadA112-P1	CTGGTGTTTTCCACCTTCCAGGGAAATGCAATTTGGCGAGTGTAGGCTGGAGCTGCTTC
aadA112-P2	CGCTGGTTCATAAATCCCCTGACAAATGTCCTCTCTTAGCATATGAATATCCTCCTTAGTTCC
aadA182-P1	CCCCTCGACCTTTGGCAATCCACGGCAGATGTGCAGGGAGTGTAGGCTGGAGCTGCTTC
aadA182-P2	TACCAGATACGCGCCAGGGTTAAAACGATATGATACTCATATGAATATCCTCCTTAGTTCC
aadA192-P1	TGTGCAGGGAGATGAGTATCATATCGTTTTAACCCTGGCGTGTAGGCTGGAGCTGCTTC
aadA192-P2	TTAGAGGTAAATCTCCCGGTAGAAAGGGTATACCAGATACATATGAATATCCTCCTTAGTTCC
aadA205-P1	GCGTATCTGGTATACCCTTTCTACCGGGAGATTTACCTCTGTGTAGGCTGGAGCTGCTTC
aadA205-P2	CTTCTGGCAACTGAGGTAACAGCCAGTCAGCCGCCGCATCATATGAATATCCTCCTTAGTTCC
Oligos for replacing the cat-sacB-T0 cassettes and introducing point mutations[b]:	
aadA_E87Q	CCAGGGAACCAGCTGCGAATAAAGCACGACAGTGACCTGCAGAGCGCGTTTTTCTGCCGACGCCCCCGGCGGCGA
aadA_E87A	CCAGGGAACCAGCTGCGAATAAAGCACGACAGTGACCGCCAGAGCGCGTTTTTCTGCCGACGCCCCCGGCGGCGA
aadA_W112F	TGGTTCATAAATCCCCTGACAAATGTCCTCTCTTAGGAACTCGCCAAATTGCATTTCCCTGGAAGGTGGAAAACA
aadA_W112A	TGGTTCATAAATCCCCTGACAAATGTCCTCTCTTAGCGCCTCGCCAAATTGCATTTCCCTGGAAGGTGGAAAACA

aadA_D182N	CCAGATACGCGCCAGGGTTAAAACGATATGATACTCATTTCCCTGCACATCTGCCGTGGATTGCCAAAGGTCGAG
aadA_D182A	CCAGATACGCGCCAGGGTTAAAACGATATGATACTCAGCTCCCTGCACATCTGCCGTGGATTGCCAAAGGTCGAG
aadA_R192A	AGAGGTAAATCTCCCGGTAGAAAGGGTATACCAGATAGCCGCCAGGGTTAAAACGATATGATACTCATCTCCCTG
aadA_K205A	TGGCAACTGAGGTAACAGCCAGTCAGCCGCCGCATCCGCAGAGGTAAATCTCCCGGTAGAAAGGGTATACCAGAT
Primers for PCR and sequencing of the chromosomal aadA locus:	
aadAverF	GCCGTTATGCCATATTTCTG
aadAverR	ACTTCAGCGATGAGAACCTA
Primers for gap-repair cloning of mutant aadA alleles:	
aadA_grc-r	GCGACAGCGGAACCATATAAA
aadA_grc-f	ATATTTTGCTGCCTGCGGTC

[a] The 20 or 24 nt in bold font indicate the 3’ priming end, complementary to the template. The 40 underlined nt indicate the 5’-extensions used as homology for λ-red recombineering.

[b] The oligos are designed to invade during lagging strand synthesis, and are complementary to the coding strand

of aadA. The sequences complementary to the target codons are indicated with bold font.

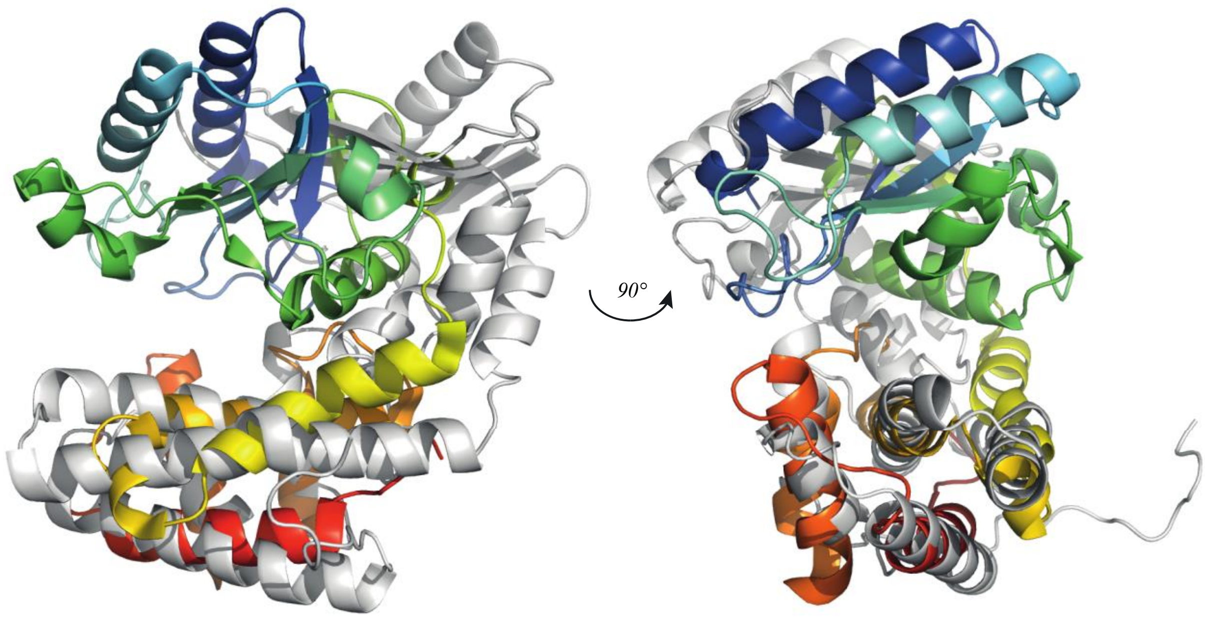

Figure S1 Superposition of AadA (rainbow colors) and KNTase (grey, pdb 1kny (Pedersen *et al.*, 1995)) based on the N-terminal domain.

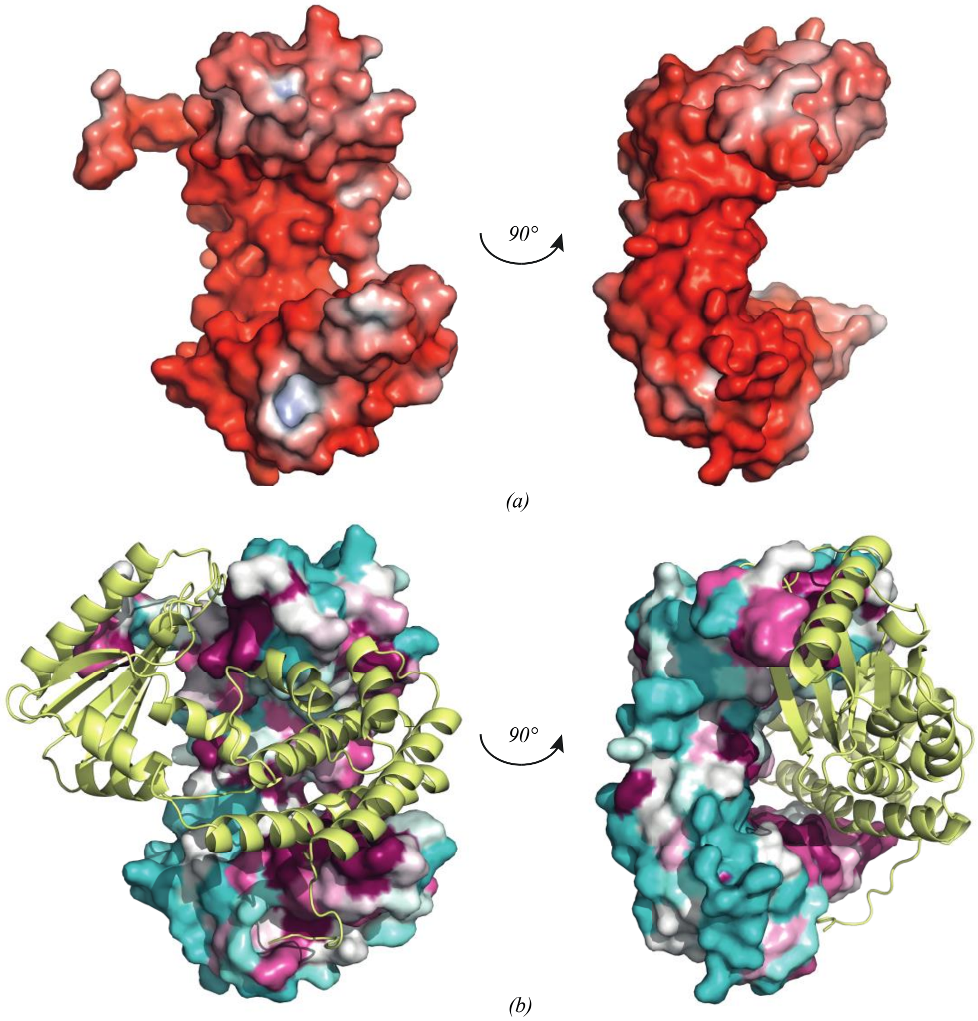

Figure S2 Surface properties of KNTase (pdb 1kny (Pedersen *et al.*, 1995)). (a) Electrostatic surface potential. The color spectrum ranges from deep red (-7 kT) to deep blue (+7 kT). (b) Surface conservation mapped by ConSurf (Ashkenazy *et al.*, 2010, Celniker *et al.*, 2013). The color spectrum ranges from magenta (highest conservation) to cyan (lowest conservation). The second subunit of KNTase is shown as cartoon in light yellow.

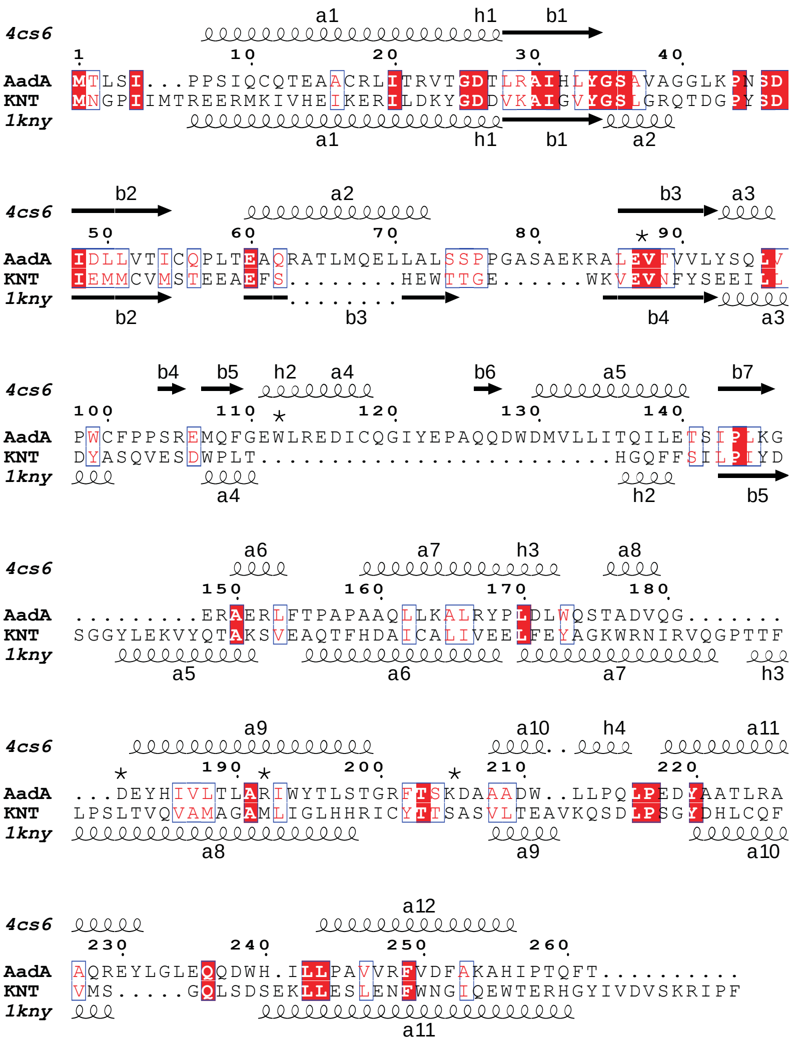

Figure S3 Structure-based sequence alignment of AadA and KNTase (pdb, 1kny (Pedersen *et al.*, 1995)). Strictly conserved residues are highlighted in red and conservative substitutions are in red font. Secondary structure is shown for AadA above the sequences and for KNTase below the sequences. Residues subjected to mutagenesis are indicated with asterisks above the alignment.

.

Supplementary references

Ashkenazy, H., Erez, E., Martz, E., Pupko, T. & Ben-Tal, N. (2010). *Nucleic Acids Res.* **38**, W529-533.

Celniker, G., Nimrod, G., Ashkenazy, H., Glaser, F., Martz, E., Mayrose, I., Pupko, T. & Ben-Tal, N. (2013). *Isr J Chem* **53**, 199-206.

Pedersen, L. C., Benning, M. M. & Holden, H. M. (1995). *Biochemistry (Mosc).* **34**, 13305-13311.
